# P27 Bacterial coinfection and antibiotic stewardship in patients with COVID-19: a systematic review and meta-analysis

**DOI:** 10.1093/jacamr/dlac004.026

**Published:** 2022-02-16

**Authors:** Maria Calderon, Grace Gysin, Sarah Walpole, Felicity Mills, Daniel Comandé, Ewan Hunter

**Affiliations:** Universidad Peruana Cayetano Heredia, Avenida Honorio Delgado 430 Urb. Ingeniería, Lima 31, San Martín de Porres 15102, Peru; London School of Hygiene & Tropical Medicine, Keppel St, Bloomsbury, London, WC1E 7HT, UK; Department of Infection and Tropical Medicine, Newcastle upon Tyne Hospitals NHS Foundation Trust, Royal Victoria Infirmary, Queen Victoria Rd. NE1 4LP, UK; Greater Glasgow and Clyde NHS Trust, 1055 Great Western Road, Glasgow, G12 0XH, UK; Institute of Clinical Effectiveness and Health Policy, Emilio Ravignani 2024 (C1414CPV), Buenos Aires, Argentina; Department of Infection and Tropical Medicine, Newcastle upon Tyne Hospitals NHS Foundation Trust, Royal Victoria Infirmary, Queen Victoria Rd. NE1 4LP, UK

## Abstract

**Introduction:**

Understanding the proportion of patients with COVID-19 who have respiratory bacterial coinfections and the responsible pathogens is important to managing COVID-19 effectively while ensuring responsible antibiotic use.

**Objectives:**

To estimate the frequency of bacterial coinfection in COVID-19 patients and of antibiotic prescribing and to appraise the use of antibiotic stewardship criteria.

**Methods:**

Systematic review and meta-analysis was performed using major databases up to May 15, 2020. We included studies that reported a) proportion/prevalence of bacterial coinfection in COVID-19 patients and b) use of antibiotics. Where available, data on duration and type of antibiotics, adverse events, and any information about antibiotic stewardship policies were also collected.

**Results:**

We included 39 studies with a total of 10 815 patients. The overall prevalence of bacterial coinfection was 10.6% (95% CI 6.8%–14.3%). When only confirmed bacterial coinfections were included the prevalence was 4.6% (95% CI 2.9%–7%). Thirty-five bacterial species were identified, the most frequent being *Mycoplasma pneumoniae* (*n = *12 [34%]), *Pseudomonas aeruginosa* (*n = *4 [11.4%]) and *Legionella pneumophila* (*n = *2 [6%]). The overall antibiotic use was 71.3% (95% CI 63.1%–79.5%). Only one study described criteria for stopping them. All included studies had a moderate to high risk of bias.

**Conclusions:**

There is currently insufficient evidence to support widespread empirical use of antibiotics in most hospitalized patients with COVID-19, as the overall proportion of bacterial coinfection is low. Furthermore, as the use of antibiotics appears currently to be largely empirical, it is necessary to formulate clinical guidelines to promote and support more targeted administration of antibiotics in patients admitted to hospital with COVID-19.

